# Sudden Knee Buckling During Gait Caused by Infarction of the Paramedian Pontine Artery: A Case Report

**DOI:** 10.7759/cureus.93485

**Published:** 2025-09-29

**Authors:** Ryo Tsujinaka, Ryo Tanaka, Hikari Suzuki, Yumi Izutani, Kaede Morimoto

**Affiliations:** 1 Department of Rehabilitation, Izumisano Yujinkai Hospital, Osaka, JPN; 2 Department of Rehabilitation, Home-Visit Station Tetoteto Izumisano, Osaka, JPN; 3 Department of Social Work and Rehabilitation Science Occupational Therapy Major, Kyoto Koka Women’s University, Kyoto, JPN

**Keywords:** cerebellar ataxia, gait, knee buckling, paramedian pontine artery infarction, physiotherapy rehabilitation

## Abstract

This case report describes a female in her 90s with an infarction of the left paramedian pontine artery (PPA) who presented with sudden knee buckling during gait. Upon admission to the convalescent rehabilitation ward, she exhibited motor paralysis, ataxia, and contralateral body lateropulsion, making standing difficult. At the interim evaluation, she was able to ambulate under supervision with a walker; however, knee buckling occurred at gait initiation and during turning. Surface electromyography of the right rectus femoris and biceps femoris during these episodes showed abnormal muscle activation. It was inferred that reduced antigravity muscle output via the vestibulospinal tract, impaired motor processing due to paresis, delayed activation timing from ataxia, and global postural control deficits collectively contributed to the knee buckling. A comprehensive physiotherapy program was implemented. Subsequently, her functional scores improved substantially from admission to discharge, including a marked increase in the Berg Balance Scale (BBS: 4 → 47) and motor items of the Functional Independence Measure-Motor domain (FIM-M: 17 →52). Motor paresis, ataxia, vestibular dysfunction, and postural control deficits improved, and knee buckling resolved. To our knowledge, this is the first report indicating that PPA infarction can cause sudden knee buckling during gait.

## Introduction

Pontine infarction causes diverse impairments, including motor paresis, sensory deficits, dysarthria, ataxia, ocular motor dysfunction, and vertigo, with a home discharge rate as low as 34.3%, indicating a significant impact on functional outcomes [1,2]. Few reports have described the clinical course of paramedian pontine artery (PPA) infarction in the context of rehabilitation. PPA infarction is associated with motor paresis and cerebellar ataxia; when accompanied by impaired consciousness, prognosis is poor. However, early diagnosis and appropriate treatment have been linked to favorable recovery [3,4]. The medial pons contains descending tracts, including the reticulospinal pathway and the vestibulospinal tract, which descend from the vestibular nuclei to regulate posture and antigravity functions. Therefore, infarction in the PPA territory may disrupt these tracts, leading to impaired lower limb antigravity muscle activity and postural instability.

Stroke significantly impairs gait ability. Gait abnormalities such as knee hyperextension or knee buckling of the paretic lower limb during the stance phase are common post stroke [5,6], typically attributed to motor paresis and altered muscle activation patterns [5]. Among patients in acute stroke units, only 39% regain independent walking by three months [7]. Conversely, 60% of those treated in rehabilitation wards achieve independent ambulation within the same period, highlighting the importance of targeted rehabilitation. Importantly, most reported gait disturbances are linked to motor paresis associated with supratentorial stroke, and limited evidence exists on the potential contribution of other clinical features, particularly ataxia resulting from infratentorial lesions.

Rehabilitation programs incorporating static and dynamic balance training, along with coordination exercises, have been shown to improve outcomes in patients with postural instability caused by cerebellar ataxia. Improvements include reduced scores on the Scale for the Assessment and Rating of Ataxia (SARA), increased gait speed, and gains on the Functional Independence Measure (FIM^TM^, version 3.0; Netsmart Technologies, Inc., Overland Park, KS) [8] and Berg Balance Scale (BBS) [9]. A recent systematic review on physiotherapy for degenerative cerebellar ataxia found that multifaceted interventions targeting balance, aerobic capacity, strength, coordination, gait, and activities of daily living (ADL) significantly decreased SARA scores and improved motor function [10]. However, no studies have specifically examined rehabilitation outcomes in patients with PPA infarction.

We report the case of an older adult with PPA infarction who exhibited knee buckling during gait, which impaired walking. Through a multifaceted physiotherapy approach emphasizing motor and postural control, the patient ultimately regained functional ambulation.

## Case presentation

**Figure 1:**
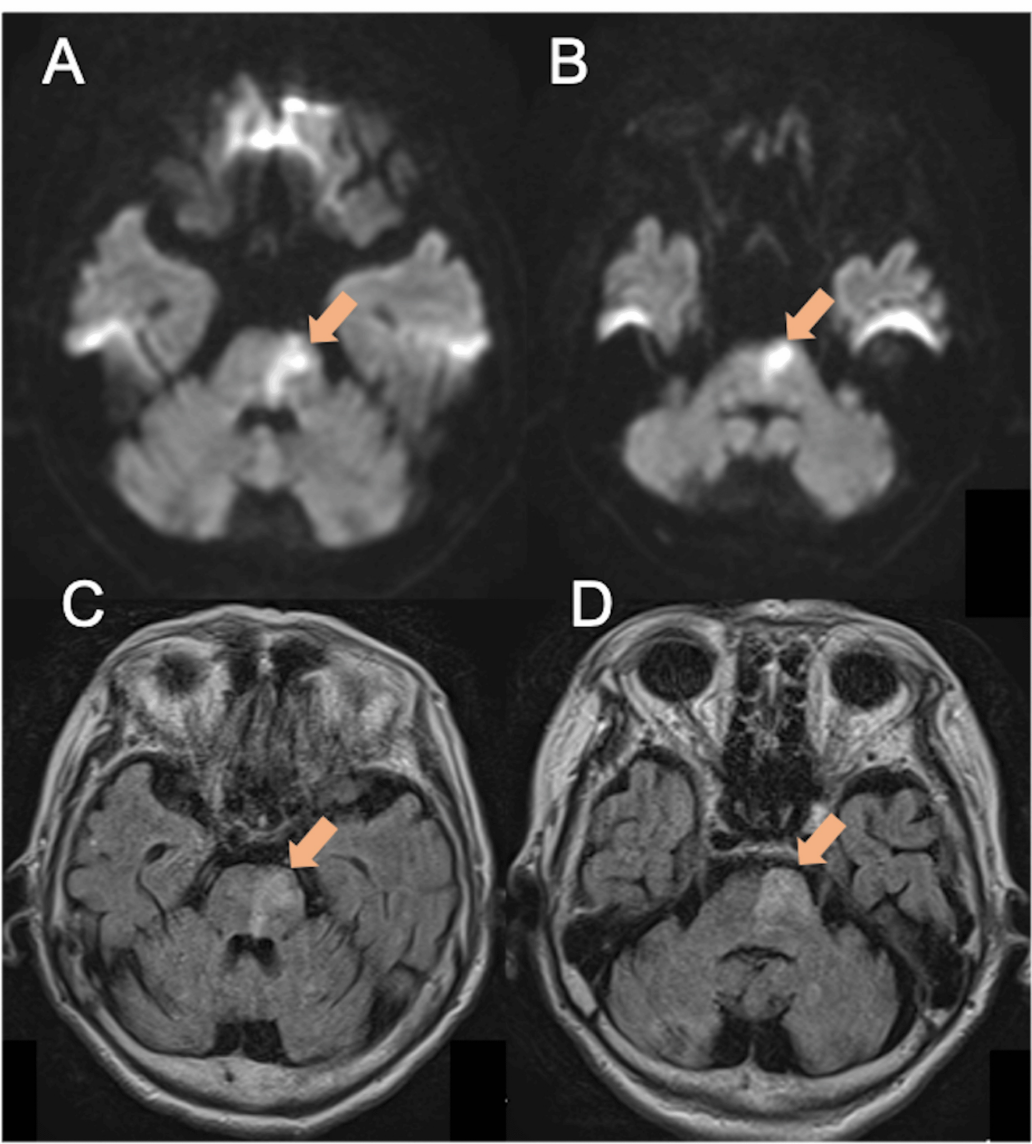
Magnetic resonance imaging (MRI) at symptom onset, including diffusion-weighted imaging (DWI: A,B) and fluid-attenuated inversion recovery (FLAIR: C,D) sequences The infarction was located in the left paramedian artery territory and involved the corticospinal tract, pontocerebellar fibers, medial lemniscus, reticular formation, superior cerebellar peduncle, and vestibular nuclei.

The patient was a 92‑year‑old female whose ADL had been independent prior to onset. She ambulated independently using a walker. However, she had a history of multiple hospitalizations due to falls, including hip fractures and vertebral compression fractures, and was considered frail. While residing in a nursing facility, she developed dysarthria and incomplete right hemiparesis and was transported to an acute-care hospital. Upon admission, her Japan Coma Scale score was 2, and magnetic resonance imaging revealed an atherothrombotic infarction in the left paramedian pontine artery territory (Figure 1).

She was admitted for medical management the same day, and conservative treatment was provided in the acute-care hospital. Although no infarct progression occurred during the acute phase, incomplete right hemiparesis resulted in significant functional decline, and she required total assistance. On day 24, she was transferred to a convalescent rehabilitation ward and began daily intensive rehabilitation comprising approximately three hours of physiotherapy, occupational therapy, and speech‑language therapy. Occupational and speech-language therapy focused on ADL training, including toileting, and on improving swallowing function.

At admission, she required moderate assistance for bed mobility and transfers, and mild assistance for sitting. She had not been diagnosed with dementia. Furthermore, the patient demonstrated good motivation and compliance with rehabilitation and actively engaged in the therapy. Physiotherapy assessments from admission to discharge are summarized in Table 1.

**Table 1:** Physical therapy assessment T1: 1 month from admission, T2: 2 month from admission, T3: 3 month from admission, FMA-LE: Fugl-Meyer assessment lower extremity (total score: 34), TCT: trunk control test (total score: 100), BBS: Berg balance scale (total score: 54), BLS: Burke lateropulsion scale (total score: 16), SARA: scale for the assessment and rating of ataxia (total score: 40), TAT: trunk ataxic test (stages I-Ⅳ), BT: bucket test, FIM-M: functional independence measure-motor domain (total score: 91)

Domain	Scale	Admission	T1	T2	T3	Discharge
Motor function	FMA-LE	24	29	28	28	31
	TCT	0	62	100	100	100
Balance	BBS	4	13	41	43	47
	Minibestest	-	0	5	3	4
	BLS	4	‐	2	0	0
Ataxia	SARA	26.5	17.5	‐	12.5	11
	TAT	II	I	I	I	I
Vestibular function	BT	‐	‐	5°	‐	0°
Activities of daily living	FIM-M	17	28	34	50	52

**Figure 2:**
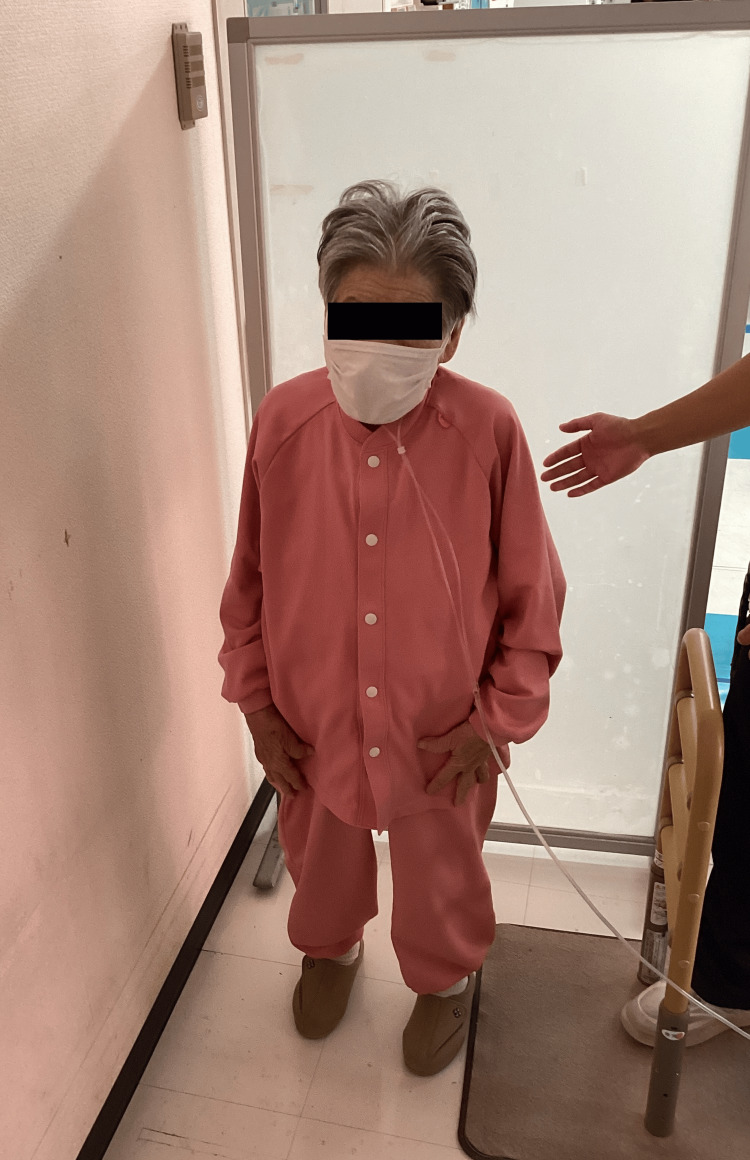
Perception of body tilt through wall contact during standing A task in which the individual stands alongside a wall and perceives body tilt through tactile feedback when the body leans to the right and comes into contact with the wall.

Standing was difficult due to body lateropulsion (BL, an involuntary tendency to fall to one side) contralateral to the lesion, noted from the initial evaluation. The bucket test indicated a 5° rightward tilt of the subjective visual vertical. When standing next to a wall, contact on the right side allowed her to perceive this tilt (Figure 2).

**Figure 3:**
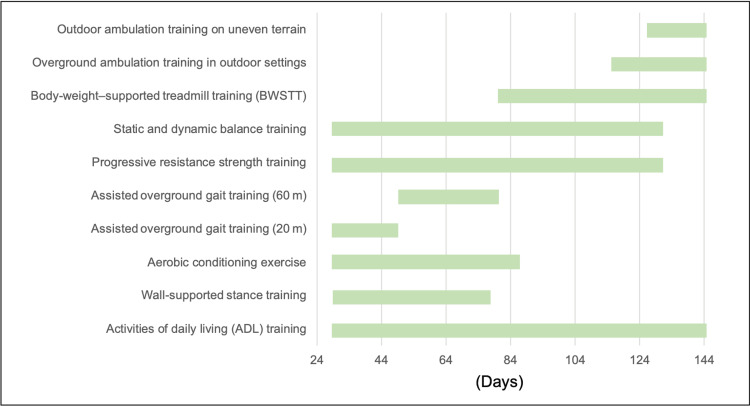
Physical therapy intervention

Physiotherapy interventions are outlined in Figure 3.

On day 38, her peripheral oxygen saturation (SpO₂) declined from 98% at rest to 90-89% during activities at 1.5-1.8 METs. Supplemental oxygen (1 L/min) was introduced during therapy and while out of bed for meals or toileting. Activity intensity was gradually increased, and oxygen flow was adjusted between 0 and 2 L/min according to SpO₂ fluctuations. Oxygen support was discontinued on day 56. During walker training, she ambulated 20 m under supervision on day 62 and 60 m without SpO₂ decline on day 71. By day 75, supervised walker ambulation was adopted throughout the ward. However, knee buckling occurred at gait initiation and during turning. She described the episodes as frightening, stating, “my leg suddenly loses power.”

**Figure 4:**
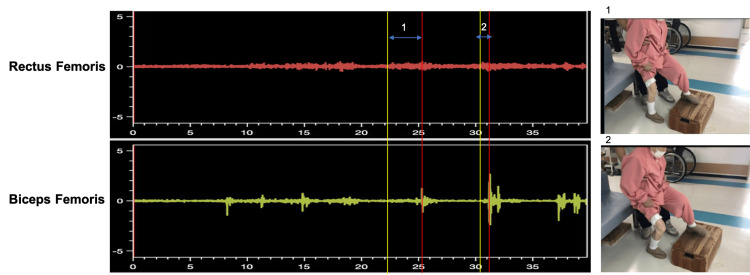
Electromyographic recordings of the right rectus femoris and right biceps femoris during the step-up task with the left lower limb Yellow line (A1): onset of muscle activity; red line (A2): left limb contact with the step. Condition 1: non-buckling trials; Condition 2: buckling trials

To investigate the cause of knee buckling, surface electromyography (EMG; TS‑MYO, Trunk Solution, Japan) of the right rectus femoris and biceps femoris was recorded during elevation of the left limb onto a step, which reliably provoked right knee buckling. EMG recordings were limited to the rectus femoris and biceps femoris because these muscles are considered to contribute substantially to knee buckling and postural stability during gait. EMG signals were processed using a 450 Hz low-pass filter, a 50 Hz high-pass filter, and root-mean-square conversion every 50 ms. Muscle activation onset (A1) was defined as sustained activity >2 SD above baseline for ≥50 ms; endpoint (A2) was when the left foot fully contacted the step, at which point vibration artifacts appeared in the biceps femoris trace. EMG data are shown in Figure 4.

The rectus femoris showed no notable changes. However, the duration from A1 to A2 for the biceps femoris was 2,754 ms when buckling did not occur, but was shortened to 88 ms during buckling episodes, representing a reduction of approximately 97%. These findings suggested that muscle activation was insufficient before single-limb support began, and the postural response was delayed.

**Figure 5:**
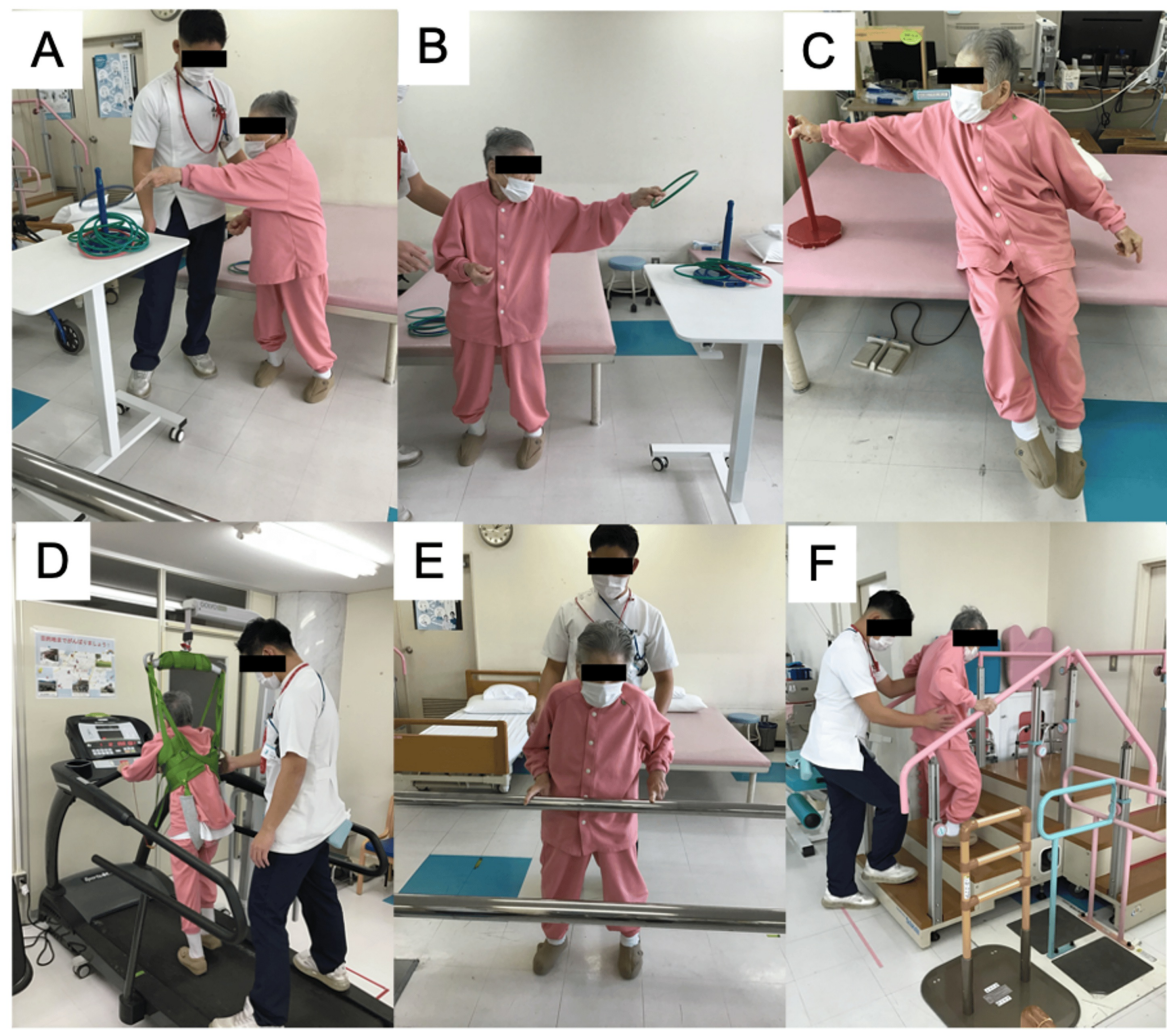
Content of physical therapy interventions Representative physical therapy interventions applied in this case, including balance, trunk, and gait training tasks.

Based on EMG findings and physiotherapy assessments, knee buckling was attributed to delayed activation of antigravity extensors due to ataxia, compounded by motor paresis and global postural control deficits, as evidenced by the Mini-Balance Evaluation Systems Test (Mini-BESTest) [11,12]. In the anticipatory postural adjustment subitem, backward swaying occurred during unsupported standing. When she lightly touched the bed rail instead of grasping it, movement stabilized, suggesting that tactile input compensated for vestibular and anticipatory control deficits rather than improving lower-limb extensor strength. Furthermore, reactive postural control was absent during standing sway, and she fell vertically, requiring assistance during sit-to-stand transitions. Therefore, a multifaceted physiotherapy program was implemented, including balance tasks that provoked knee buckling, strengthening exercises, and body-weight-supported treadmill training (Figure 5).

By day 118, knee buckling episodes decreased, and she achieved independent walker ambulation within the ward during the day. Outdoor ambulation began on day 126, and she was able to walk approximately 50 m on level ground under supervision, without knee buckling - even on uneven surfaces. By day 141, she ambulated independently with a walker throughout the day. The BBS improved from 4 at admission to 47 at discharge, reflecting a substantial reduction in fall risk and enabling independence in tasks such as toileting and transfers. Consequently, the motor items of the FIM-M also showed a marked improvement, increasing from 17 to 52. Discharge to the nursing facility was scheduled on day 148.

## Discussion

Knee buckling during gait following stroke is typically attributed to decreased activity in the soleus, tibialis anterior, quadriceps, and hamstrings [5]. Knee hyperextension is a common compensatory mechanism post stroke, as it shifts the ground reaction force vector closer to the center of mass, enhancing stability [13]. In this case, such compensation through hyperextension was inconsistently applied, and knee buckling occurred. Episodes often appeared during turning or left limb elevation, likely reflecting increased load on the right limb. However, the phenomenon was not consistently reproducible. From a biomechanical perspective, when knee hyperextension stabilizes gait, the hamstrings play a key role in joint control [14]. Therefore, delayed activation of the biceps femoris during these episodes may have failed to provide adequate stabilization, resulting in knee buckling.

Gait in individuals with cerebellar ataxia is characterized by reduced walking speed, shorter step length, increased stride-time variability, prolonged stance and swing phases, and increased step-width variability [15,16]. However, none of these studies reported the specific knee buckling observed in this case. Prior electromyographic research on ataxic gait demonstrated delayed biceps femoris activation compared to controls [17], suggesting that latency in hamstring recruitment may contribute to limb collapse. Still, one study comparing patients with impaired balance to those with impaired voluntary leg coordination found that balance impairment was associated with reduced peak flexion angles at the hip, knee, and ankle [16]. These findings imply that, in isolated ataxia, compensatory joint stiffening may prevent knee buckling.

In this patient, motor paresis, vestibular dysfunction, and general postural control deficits coexisted with ataxia. Regarding motor paresis, studies have shown that muscle contraction in response to stimuli is delayed in patients with hemiparesis compared to healthy individuals [18]. Additionally, paresis can diminish activation of antigravity muscles during gait, including the soleus, tibialis anterior, quadriceps, and hamstrings [5]. Vestibular dysfunction may also contribute. Galvanic vestibular stimulation has been shown to increase H-reflex amplitude in upright postures, indicating that excitability of the vestibulospinal tract affects antigravity muscle output [19]. Early-phase body lateropulsion (BL) in this patient likely resulted from damage to ascending graviceptive pathways, which cross above the vestibular nuclei and ascend through the paramedian tegmentum to the thalamus and cortical regions [20].

These findings suggest that delayed muscle activation due to impaired postural control and ataxia was a primary factor in the patient’s knee buckling. Although joint stiffening was attempted as a compensatory strategy, mild motor paresis and vestibular dysfunction likely reduced the strength and timing of antigravity extensor activation. In supratentorial strokes, knee hyperextension commonly occurs as a result of reduced muscle output or muscle overactivity due to motor paresis [14]. In contrast, in pontine lesions with cerebellar ataxia, such as the present case, compensatory strategies using knee hyperextension may be limited not only by motor paresis but also by the overlapping presence of ataxia, vestibular dysfunction, and general postural control deficits, which may ultimately lead to sudden knee buckling. A multifaceted physiotherapy approach - previously shown to improve outcomes in patients with ataxia [9,10] - was applied to address these overlapping impairments. Among the interventions, body-weight supported treadmill training (BWSTT) initiated at the time knee buckling became a clinical concern was most likely to have contributed to muscle activation timing in motor control during gait. Improvements in motor paresis, ataxia, vestibular function, and global postural control corresponded with the resolution of knee buckling.

A major limitation of this case report is the absence of electromyographic recordings from the contralateral (non-buckling) limb. Therefore, it remains unclear whether biceps femoris activation was intrinsically delayed in the buckling limb, even during strides without knee buckling. Moreover, because knee buckling occurred suddenly and unpredictably, systematic confirmation of EMG findings across repeated trials was not possible, and the reproducibility of the abnormal activation patterns could not be fully established. Given that these findings are based on a single patient, further studies involving larger cohorts and bilateral surface EMG assessments are necessary to clarify inter-limb differences in hamstring activation timing during gait.

## Conclusions

We report a patient with sudden knee buckling during gait due to infarction of the paramedian pontine artery. To our knowledge, detailed reports describing the rehabilitation course of patients with PPA infarction are scarce. In addition to the established clinical features of motor paresis, ataxia, and vestibular dysfunction, this case demonstrates that PPA infarction can cause knee buckling during gait.
